# Supplementary material for: Age-appropriate vaccination and associated factors among children aged 12–35 months in Ethiopia: A multi-level analysis
Source: PLoS One. 2024 Oct 10;19(10):e0305801. doi: 10.1371/journal.pone.0305801 (PMC11466417; doi:10.1371/journal.pone.0305801)
Supplement: Attachment — Submitted filename: PONE-D-24-22259_reviewer.pdf [file pone.0305801.s001.pdf]

PLOS ONE

Age-appropriate Vaccination and Associated Factors among Children Aged 12- 35 Months in Ethiopia: A Multi-Level Analysis

--Manuscript Draft--

**Manuscript Number:**	PONE-D-24-22259
**Article Type:**	Research Article
**Full Title:**	Age-appropriate Vaccination and Associated Factors among Children Aged 12- 35 Months in Ethiopia: A Multi-Level Analysis
**Short Title:**	Age-appropriate Vaccination among Children Aged 12- 35 Months in Ethiopia
**Corresponding Author:**	Ebissa Bayana<br>Jimma University<br>Jimma, ETHIOPIA
**Keywords:**	Age-appropriate, Immunization, Vaccinations, Ethiopia.
**Abstract:**	Background: Age-appropriate vaccination is one of the key public health measures to prevent morbidity and mortality worldwide. Despite its importance, there has been insufficient emphasis on tackling this problem. Therefore, this study aimed to determine the prevalence of age-appropriate vaccination and associated factors in Ethiopia.<br>Method: Data of 1077 children aged 12-35months were extracted from the Ethiopian Mini Demographic and Health Survey 2019 using a prepared data extraction checklist and included in the analysis. The extracted data was analyzed using STATA version 14.0. Descriptive and inferential statistics were applied. then analysis at different levels was done using multilevel logistic regression. Significant variables were identified at p-value < 0.05 within 95% confidence level and AOR.<br>Result: The pooled prevalence of age-appropriate vaccination in this study was 21.17% at 95%CI (18.73-23.61). Factors like mothers aged >= 40 (AOR=4.05 at 95%CI1.03, 15.83), 35-39 years (AOR= 4.62 at 95%CI1.27,16.71), 25-29 years, (AOR =4.07 at 95%CI 1.18,14.03), Maternal secondary education (AOR=1.85 at 95% CI 1.06, 3.22), Maternal primary education (AOR= 1.60 at 95% CI1.07, 2.41) and rural residence (AOR=0.34, 95%CI 0.23,0.51) were significant predictors of age-appropriate vaccinations.<br>Conclusion:This study concluded that the prevalence of age-appropriate vaccination of children in Ethiopia is below the desired level. Hence, the stakeholders should give due attention to the timely vaccination of children equally as the effort being made to increase the coverage.
**Order of Authors:**	Bekelu Teka Worku
	Eshetu Alemayehu Wordofa
	Gadisa Senbeto
	Beakal Zinab
	Ebissa Bayana Kebede
	Fira Abamecha
	Gurmessa Tura Debela
	Negalign Birhanu
	Yibeltal Siraneh
	Dessalegn Tamiru
**Additional Information:**	
**Question**	**Response**
**Financial Disclosure**<br><br>Enter a financial disclosure statement that	The author(s) received no specific funding for this work.

describes the sources of funding for the work included in this submission. Review the submission guidelines for detailed requirements. View published research articles from *PLOS ONE* for specific examples.<br><br>This statement is required for submission and **will appear in the published article** if the submission is accepted. Please make sure it is accurate.<br><br>**Funded studies**<br>Enter a statement with the following details:<br>• Initials of the authors who received each award<br>• Grant numbers awarded to each author<br>• The full name of each funder<br>• URL of each funder website<br>• Did the sponsors or funders play any role in the study design, data collection and analysis, decision to publish, or preparation of the manuscript?<br><br>Did you receive funding for this work?	
**Competing Interests**<br><br>Use the instructions below to enter a competing interest statement for this submission. On behalf of all authors, disclose any competing interests that could be perceived to bias this work—acknowledging all financial support and any other relevant financial or non-financial competing interests.<br><br>This statement is required for submission and **will appear in the published article** if the submission is accepted. Please make sure it is accurate and that any funding sources listed in your Funding Information later in the submission form are also declared in your Financial Disclosure statement.<br><br>View published research articles from *PLOS ONE* for specific examples.	The authors have declared that no competing interests exist

**NO authors have competing interests**<br><br>Enter: *The authors have declared that no competing interests exist.*<br><br>**Authors with competing interests**<br><br>Enter competing interest details beginning with this statement:<br><br>*I have read the journal's policy and the authors of this manuscript have the following competing interests: [insert competing interests here]*<br><br>* typeset	
**Ethics Statement**<br><br>Enter an ethics statement for this submission. This statement is required if the study involved:<br><br>• Human participants<br>• Human specimens or tissue<br>• Vertebrate animals or cephalopods<br>• Vertebrate embryos or tissues<br>• Field research<br><br>Write "N/A" if the submission does not require an ethics statement.<br><br>General guidance is provided below. Consult the submission guidelines for detailed instructions. **Make sure that all information entered here is included in the Methods section of the manuscript.**	For the data we requested www.DHSmeasure.org, to obtain permission to access the data set from https://www.dhsprogram.com/data/available-datasets.cfm by submitting abstract. The DHS public-use datasets are protected from identification of respondents, households, or sample communities by procedures approved by the Institution Review Board. The data files do not contain household addresses or names of individuals. The data set was not used for other purposes except for the intended objective.

Powered by Editorial Manager® and ProduXion Manager® from Aries Systems Corporation

**Format for specific study types**<br><br>**Human Subject Research (involving human participants and/or tissue)**<br>• Give the name of the institutional review board or ethics committee that approved the study<br>• Include the approval number and/or a statement indicating approval of this research<br>• Indicate the form of consent obtained (written/oral) or the reason that consent was not obtained (e.g. the data were analyzed anonymously)<br><br>**Animal Research (involving vertebrate animals, embryos or tissues)**<br>• Provide the name of the Institutional Animal Care and Use Committee (IACUC) or other relevant ethics board that reviewed the study protocol, and indicate whether they approved this research or granted a formal waiver of ethical approval<br>• Include an approval number if one was obtained<br>• If the study involved *non-human primates*, add *additional details* about animal welfare and steps taken to ameliorate suffering<br>• If anesthesia, euthanasia, or any kind of animal sacrifice is part of the study, include briefly which substances and/or methods were applied<br><br>**Field Research**<br><br>Include the following details if this study involves the collection of plant, animal, or other materials from a natural setting:<br>• Field permit number<br>• Name of the institution or relevant body that granted permission	
**Data Availability**<br><br>Authors are required to make all data underlying the findings described fully available, without restriction, and from the time of publication. PLOS allows rare exceptions to address legal and ethical concerns. See the PLOS Data Policy and FAQ for detailed information.	Yes - all data are fully available without restriction

A Data Availability Statement describing where the data can be found is required at submission. Your answers to this question constitute the Data Availability Statement and **will be published in the article**, if accepted.<br><br>**Important:** Stating 'data available on request from the author' is not sufficient. If your data are only available upon request, select 'No' for the first question and explain your exceptional situation in the text box.<br><br>Do the authors confirm that all data underlying the findings described in their manuscript are fully available without restriction?	
**Describe where the data may be found in full sentences. If you are copying our sample text, replace any instances of XXX with the appropriate details.**<br><br>• If the data are **held or will be held in a public repository**, include URLs, accession numbers or DOIs. If this information will only be available after acceptance, indicate this by ticking the box below. For example: *All XXX files are available from the XXX database (accession number(s) XXX, XXX.*).<br>• If the data are all contained **within the manuscript and/or Supporting Information files**, enter the following: *All relevant data are within the manuscript and its Supporting Information files.*<br>• If neither of these applies but you are able to provide **details of access elsewhere**, with or without limitations, please do so. For example:<br><br>*Data cannot be shared publicly because of [XXX]. Data are available from the XXX Institutional Data Access / Ethics Committee (contact via XXX) for researchers who meet the criteria for access to confidential data.*<br><br>*The data underlying the results presented in the study are available from (include the name of the third party*	The data used for this study is included within the article itself

and contact information or URL).<br>• This text is appropriate if the data are owned by a third party and authors do not have permission to share the data.<br><br>* typeset	
Additional data availability information:	

Manuscript

Click here to access/download;Manuscript;Manuscript_Vaccination.docx

Age-appropriate Vaccination and Associated Factors among Children Aged 12- 35 Months in Ethiopia: A Multi-Level Analysis

Bekelu Teka Worku[1¶], Eshetu Alemayehu Wordofa[5¶,] Gadisa Senbeto[2¶], Beakal Zinab[3¶], Ebissa Bayana Kebede[4*¶], Fira Abamecha[6¶], Gurmessa Tura Debela[1¶], Negalign Birhanu[7¶], Yibeltal Siraneh[7¶], Dessalegn Tamiru[3¶]

Authors' Affiliation

[1]Department of Population and Family Health, Jimma University, Ethiopia

[2]Department Environmental Health, Jimma University, Ethiopia

[3]Department Nutrition and Dietetics, Jimma University, Ethiopia

[4]School of Nursing, Jimma University, Ethiopia

[5]Department Epidemiology, Jimma University, Ethiopia

[6]Department of Health Behavior and Society, Jimma University, Ethiopia

[7]Department of Health Policy and Management, Jimma University and Ethiopia

Corresponding Author:

ebisabayana@ymail.com (EBK)

Abstract

Background: Age-appropriate vaccination is one of the key public health measures to prevent morbidity and mortality worldwide. Despite its importance, there has been insufficient emphasis on tackling this problem. Therefore, this study aimed to determine the prevalence of age-appropriate vaccination and associated factors in Ethiopia.

Method: Data of 1077 children aged 12-35months were extracted from the Ethiopian Mini Demographic and Health Survey 2019 using a prepared data extraction checklist and included in the analysis. The extracted data was analyzed using STATA version 14.0. Descriptive and inferential statistics were applied. then analysis at different levels was done using multilevel logistic regression. Significant variables were identified at p-value < 0.05 within 95% confidence level and AOR.

Result: The pooled prevalence of age-appropriate vaccination in this study was 21.17% at 95%CI (18.73-23.61). Factors like mothers aged >= 40 (AOR=4.05 at 95%CI1.03, 15.83), 35-39 years (AOR= 4.62 at 95%CI1.27,16.71), 25-29 years, (AOR =4.07 at 95%CI 1.18,14.03), Maternal secondary education (AOR=1.85 at 95% CI 1.06, 3.22), Maternal primary education (AOR= 1.60 at 95% CI1.07, 2.41) and rural residence (AOR=0.34, 95%CI 0.23,0.51) were significant predictors of age-appropriate vaccinations.

Conclusion: This study concluded that the prevalence of age-appropriate vaccination of children in Ethiopia is below the desired level. Hence, the stakeholders should give due attention to the timely vaccination of children equally as the effort being made to increase the coverage.

Keywords: Age-appropriate, Immunization, Vaccinations, Ethiopia.

Introduction

Vaccination involves exposing children to a weakened or killed version of a pathogen. This provokes a response in the immune system without causing serious infections (1). Immunization is a crucial public health intervention that can help to reduce child morbidity and mortality rates and increase life expectancy (2). The most common vaccine-preventable diseases among children include measles, diphtheria, influenza, tetanus, pertussis, hepatitis, mumps, pneumonia, polio, rotavirus, and others (3, 4).

To fight those vaccine-preventable diseases, the Expanded Program on Immunization (EPI) was launched by the World Health Organization (WHO) in 1974 with the main aim of providing immunization to all children worldwide. In 1984, the WHO standardized a vaccination schedule for six vaccines, namely Bacillus Calmette-Guérin (BCG), diphtheria-tetanus-pertussis (DTP), oral polio, and measles (5, 6). Similarly, Ethiopia launched an immunization program in 1980 as the EPI. The EPI was established to achievea 100% immunization rate in 10 years even though the original target remained unachieved after 20 years ~~also~~ (7, 8).

It is crucial to ensure that children receive vaccinations for their age to safeguard them from vaccine-prevalent diseases (9). Moreover, ensuring universal vaccination through routine and catch-up vaccination schedules is an essential part of quality healthcare in nations. It is linked to improved health outcomes, cognitive development, productivity, and cost savings (10-12). In line with this, the World Immunization Agenda 2030 recommends leaving no one behind for vaccination through increasing equitable access and use of new and existing vaccines (13). However, the burden of vaccine-preventable disease remains not easy in today’s world.

Nowadays, full vaccination of children timely at their appropriate age is a global public problem, and vaccination coverage has fallen back or stagnated (14). Timely vaccination among children ranges from 38% to 65% in the Philippines and median on-time vaccination in Sub-Saharan African countries is less than 50% (15, 16). In Ethiopia, the age-appropriate vaccination practice is less than half 43.7%, in the Afar region, nearly one-third 33.7% in Oromia Region, and 39.1% for specific vaccines in the North East Ethiopia (17-19). However, the pooled full vaccination coverage in Ethiopia is greater than 65% which is higher than all these figures indicating poor age-appropriate vaccination in different regions (20). On the other way, the magnitude of missed opportunities for routine vaccination is high 39.8% in Ethiopia despite the high value ofage-appropriatevaccinations(21).

Despite remarkable improvements in child health services utilization, age-appropriate childhood immunization is still challenging in Ethiopia (22). To avert this Ethiopia developed strategies and policies likethe Health Sector Transformation Plan (HSTP), reaching every district (RED) and sustainable outreach service (SOS) approaches and comprehensive multiyear immunization plans (cMYP)(23, 24). In general, the majority of the vaccination types are age-specific and some vaccinations could not be recommended after some point of time in children's age. It is highly important to comply with age-appropriate vaccinations to provide maximum effectiveness against vaccine-preventable diseases among children (9).

Revealing information on the age appropriateness of vaccination from national data can help to provide a more complete view of vaccination coverage in the country. The information could be essential to clarify the missing and delayed vaccination status that the stakeholders such as policymakers can utilize to address the gaps in immunization programs. Although some studies have assessed age-appropriate vaccination in Ethiopia, no study used the most recent national

data to evaluate this issue. Therefore, this study aimed to conduct a multi-level analysis to determine the level of age-appropriate vaccination status and associated factors in Ethiopia using the 2019 Ethiopia Mini Demographic and Health Survey (25).

Materials and Methods

Source of data and study design

The study was done in Ethiopia, the largest nation in the Horn of Africa. Ethiopia is the second most populous country in Africa and 11th in the world populous country. According to the United Nations Children's Fund, there are 13 million under five years of age children in Ethiopia (26). The study was done from January-February 2024. This study used secondary data from the Ethiopia Mini Demographic Survey of 2019 (EMDHS). The EMDHS was a cross-sectional study conducted on identified areas defined as enumeration areas constituting geographic areas. It is national representative data that was collected through a community-based cross-sectional survey from March 21, 2019, to June 28, 2019. The 2019 EMDHS is the second EMDHS and the fifth DHS implemented in Ethiopia. The Ethiopian Public Health Institute (EPHI) conducted the survey in collaboration with the Central Statistical Agency (CSA) and the Federal Ministry of Health (FMoH), with technical assistance from ICF and financial as well as technical support from development partners(27).

Population

This study focused on the children aged 12-35 months old that are sampled from all child vaccination-related data in Ethiopia. All 12-35 months of age children who have at least one confirmed vaccination history and whose vaccination card was seen at home or from health facilities were included in the study. The children were excluded if the dates of vaccination were inconsistent with their birthdates.

Sample Size Determination and Sampling Techniques

The study included 1077 children aged 12-35 months who are vaccinated according to EPI and their collected data available in EMDHS 2019. The 2019 EMDHS utilized the census frame from the 2019 Ethiopia Population and Housing Census (EPHC), established by the Central Statistical Agency (CSA), consisting of 149,093 census enumeration areas (EAs). Each EA, on average, covers 131 households and contains data regarding EA location, urban or rural residency, and estimated residential household count. Ethiopia's administrative structure was comprised of nine geographical regions and two administrative cities during the survey (27).

The sample design aimed at generating key indicators for the entire country, urban and rural areas individually, and for each of the nine regions and the two administrative cities. The sample selection process occurred in two stages within 21 strata, stratifying each region into urban and rural areas. In the first stage, 305 EAs (93 urban and 212 rural) were chosen with a probability proportional to EA size, while the second stage involved selecting a fixed number of 30 households per cluster using equal probability systematic selection from a newly established household listing. The survey carried out between March 21, 2019, and June 28, 2019, interviewed 8,855 women aged 15-49 from a nationally representative sample of 8,663 households, aiming to furnish estimates at national, regional, urban, and rural levels (27).

Operational Definitions

Age-appropriate Vaccination: Age-appropriate vaccination is children's vaccination timely within the WHO-recommended window period for basic vaccines: BCG (birth – 8 weeks), Penta1and OPV1 (6 weeks – 14 weeks); Penta2, and OPV2 (10 weeks – 18 weeks); Penta3, and OPV3 (14 weeks – 24 weeks)] and measles vaccine (9 months – 11 months) (28). Vaccination of children was coded as '1' if the child received the vaccine in the recommended period, and coded

as ‘0’ if vaccination was done before or after the recommended period. Age-appropriate vaccination was calculated from the summation of all “yes” scores for each specific basic vaccine type considered for this study.

According to the EMDHS report, the child must receive at least: one dose of BCG vaccine, which protects against tuberculosis; three doses of DPT-HepB-Hib, which protects against diphtheria, pertussis (whooping cough), tetanus, hepatitis B, and Haemophilus influenzae type b; three doses of polio vaccine; and one dose of measles vaccine (27). Therefore, we considered these vaccines in the analysis.

Data Extraction Tools and Quality Control Methods

The data extraction checklist was adapted from the EMDHS to extract necessary data. The checklist was prepared in a way to extract socio-demographics, maternal and obstetric factors, and information-related factors from the large data. Before conducting any analysis, data was extracted depending on the inclusion criteria. Then variables were identified, recoded, cleaned, computed, and missing values were coded.

Statistical Analysis

The extracted data was analyzed using the STATA version 14 statistical software. To account for differences in stratum selection and nonresponse probabilities, the data was weighted. Descriptive and inferential statistical methods were employed to present the data. Descriptive statistics such as frequency distributions and percentages were used.

A multi-level-logistic regression modeling was done to assess the association between the independent variables with the dependent variable. Variables with p-value <0.05 at 95% with AOR were declared as statistically significant using the me logit function. To check for multicollinearity among the independent variables, the variance inflation test was performed (a

default function for STATA 14.0 version) and the final model was used by comparing model 0, model I, model II, and model III. In Model 0 the dependent variable was analyzed with the regional variable which means that the initial analysis was focused on how the dependent variable is influenced by the regional variable alone. In Model I the effect of an individual-level variable on a dependent variable was analyzed which means theanalysis lookedat how a specific variable at an individual level impacts the dependent variable. Model II examined the dependent variable and the community variable which is resident. Model III level examined using significant variables in both model I and model II by the stepwise analysis method. The model with the least AIC and BIC was used to identify model fitness.

Ethical Considerations

For the data we requested www.DHSmeasure.org, to obtain permission to access the data set from https://www.dhsprogram.com/data/available-datasets.cfm by submitting abstract. The DHS public-use datasets are protected from identification of respondents, households, or sample communities by procedures approved by the Institution Review Board. The data files do not contain household addresses or names of individuals. The data set was not used for other purposes except for the intended objective.

Result

Socio-Demographic Characteristics

A total of 1077 children aged 12-35months whose vaccination card was checked at home or health facility in MEDHS 2019 were included.The mean age of mothers was 28.58 ±6.27 with a minimum of 17 years and a maximum of 49 years old. Similarly, the mean age of children was 22.34±6.70 with a minimum of 12 months and a maximum of 35 months. About half of the children were male 549 (50.97) (Table 1).

Table 1: Distribution of socio-demographic characteristics (n=1077).

Variables	Categories	Frequency	Percent
Region	Tigray	134	12.44
	Afar	52	4.83
	Amhara	117	10.86
	Oromia	135	12.53
	Somali	51	4.74
	Benishangul Gumuz	114	10.58
	SNNPR	94	8.73
	Gambela	77	7.15
	Harari	85	7.89
	Addis Ababa	110	10.21
	Diredawa	108	10.03
Place of residence	Urban	357	33.15
	Rural	720	66.85
Age of the mothers	15-19	39	3.65
	20-24	218	20.24
	25-29	390	36.21
	30-34	218	20.24
	35-39	135	12.53
	>=40	77	7.15
Mother educational status	No education	464	43.08
	Primary	399	37.05
	Secondary	120	11.14
	Higher	94	8.73

Continued ↓

Variable	Category	Frequency	Percent
Religion	Orthodox	418	38.81
	Protestant	181	16.81
	Muslim	463	42.99
	Other *	15	1.39
Sex of HH++ head	Male	888	82.45
	Female	189	17.55
Wealthy status	Poor	389	36.12
	Middle	167	15.51
	Rich	521	48.38
Marital status	currently in union/living with a man	1025	95.17
	Other **	22	4.83
Family size	Less than five	376	34.91
	Above 5	701	65.09
Number of <5 children at home	<=1	532	49.40
	>=2	545	50.60
Sex of children	Male	549	50.97
	Female	528	49.03
Age of children	Between 12-24 months	645	59.89
	Above 24 months	432	40.11

*=Catholic, Traditional, **= Single, widowed, Divorced

Maternal and obstetric-related Factors

The study revealed that almost all, 1056(98.05) births were singleton. Concerning ANC follow-upa bit higher than half, 504(55.96%) of mothers had 4 or more ANC visits. The study revealed that 745(69.17%) of mothers delivered in health facilities, while about two-thirds 456 (66.47%) of mothers had PNC follow (Table 2).

Table: 2 Distribution of maternal and Obstetric characteristics (n=1077).

Variable	Category	Frequency	Percent
Place of delivery	Health facility delivery	745	69.17
	Home delivery	332	30.83
Birth order of the child	First	270	25.07
	2-4	492	45.68
	>=5	315	29.25
Birth in the past 5 years	One	637	59.15
	Two	384	35.65
	Three or four	56	5.20
Type of birth	Single birth	1056	98.05
	Multiple births	21	1.95
ANC visit	No ANC visits	117	12.12
	Less than 4 ANC visits	308	31.92
	>=4 ANC visits	504	55.96
PNC	No	230	33.53
	Yes	456	66.47

NB: ANC= Antenatal Care, PNC= Post Natal Care

Media Exposure

The study revealed that more than half, 582 (54.04%) of the children's mothers had no access to media exposure (Fig.1).

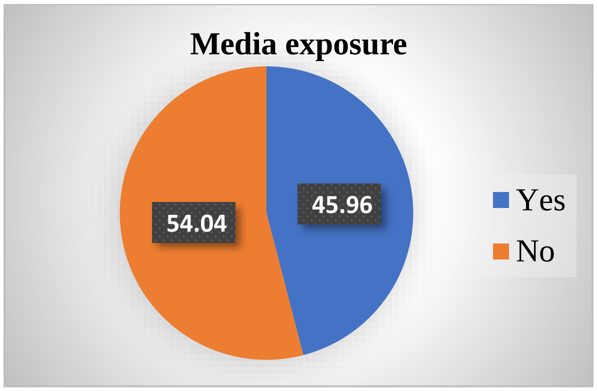

Figure 1. Distribution of children's mothers' exposure to media

Prevalence of age-appropriate Vaccination

The prevalence of age-appropriate vaccination was 21.17% 95%CI (18.73-23.61) of children who received the basic vaccinations at an appropriate age. Furthermore, theresults of the study showed that the majority of children were vaccinated for all basic vaccinations as presented in Table 3.

Table 3: Distribution of age-appropriate vaccination among children aged 12-35 months.

Basic Vaccinations	Response	Appropriate time	
		Frequency	Percent
BCG	Yes	607	62.13
	No	370	37.87
OPV1	Yes	734	69.71
	No	319	30.29
Opv2	Yes	678	68.55
	No	311	31.45
OPV3	Yes	662	71.49
	No	264	28.51
Penta1	Yes	733	69.22
	No	326	30.78
Penta 2	Yes	697	68.98
	No	309	31.02
Penta 3	Yes	670	72.28
	No	257	27.72
Measles	Yes	473	60.80
	No	305	39.20

Factors Associated With Age-appropriate Vaccination Among Children Aged 12-35months

In the multilevel logistic regression analysis, the age of mothers, educational status of the mothers, and childbirth in the past five years were statistically significant determinants of age-appropriate vaccination

The study identified that children's mothers aged >= 40 years were 4.05 times (AOR=4.05 at 95%CI1.03, 15.83) more likely to vaccinate their child at an appropriate age compared to those aged between 15-19 years old. Similarly, 35-39-year-old mothers were 4.62 times (AOR= 4.62 at 95%CI 1.27, 16.71) more likely to vaccinate their child at an appropriate age compared to those aged between 15-19 years old. In addition, 25-29-year-old mothers were 4.07 times (AOR =4.07 at 95%CI1.18, 14.03) more likely to vaccinate their child at appropriate age compared to those aged between 15-19 years old.

Regarding maternal education, mothers who completed secondary education were 1.85 times (AOR=1.85 at 95% CI1.06, 3.22) more likely to vaccinate their child at an appropriate age compared to those who had no education. In addition, mothers who completed primary education were 1.60 times (AOR= 1.60, 95% CI=1.07, 2.41) more likely vaccinated at an appropriate age compared to those who had no education.

Regarding place of residence, mothers wholive in rural areas were 66% (AOR=.34 at 95%CI.23,.51) less likely to vaccinate their child at an appropriate age compared to those who live in urban areas (Table 4).

Model fit statistics

As shown in Table 4 below, a small number of AIC and BIC was Model IV, which indicates that the model well explained the factors better than the Null Model, Models II and III. Therefore, this made the final as the best-fitted model than others.

Table 4: Multilevel logistic regression analysis of factors associated with age-appropriate vaccination among children aged 12-35 months.

**Variables**	**Category**	**Model0**	**Model I**	**Model II**	**Model III**
Age of mother	15-19	-	1	-	1
	20-24	-	1.94(.55,6.82)	-	2.01(.56,7.11)
	25-29	-	3.99(1.16,13.65)	-	4.07(1.18,14.03)*
	30-34	-	2.84(.80,10.0)	-	2.71(.76,9.63)
	35-39	-	4.87(1.36,17.47)	-	4.62(1.27,16.71)*
	>=40	-	3.96(1.02,15.31)	-	4.05(1.03,15.83)*
Place of	Health facility	-	1	-	1
delivery	Home/other	-	.53(.32,82)	-	.63(.40,.98)
Media	Yes	-	1.46(1.00,2.15)	-	1.01(.68,1.52)
Exposure	No	-	1	-	1
Educational	No education	-	1	-	1
status	Primary	-	1.73(1.15,2.59)	-	1.60(1.07,2.41)*
	Secondary	-	2.16(1.25,3.74)	-	1.85(1.06,3.22)*
	Higher	-	2.04(1.13,3.69)	-	1.59(.88,2.88)
Child birth in	One	-	1	-	1
the past 5	Two	-	.80(.56,.1.13)	-	.84(.59,1.19)
years	Three/Four	-	.76(.32,1.82)	-	.76(.32,1.84)
Religion	Orthodox	-	1	-	1
	Protestant	-	.62(.35,1.11)	-	.62(.38,1.02)
	Muslim	-	.81(.55,1.19)	-	.79(.55,1.12)
	Other	-	1.21(.29,4.91)	-	1.10(.28,4.23)
Residence	Urban	-		1	1
	Rural	-		.26(.18,.37)	.34(.23,.51)
Std err		.221623	0.8586	0.0883094	3.15e-16

Log-likelihood	-539.082	-508.2364	-512.7568	-494.962
AIC	1082.16	1050.473	1031.514	1023.924
BIC	1092.12	1135.166	1046.459	1108.617

*= statistically significant at P<0.05, 1= reference category, AIC= Akaike information criterion, BIC =Bayesian Information Criterion

Discussion

The present study aimed to assess the magnitude and factors associated with age-appropriate vaccination among children aged 12-35 months in Ethiopia. Our results showed thatthe prevalence of age-appropriate vaccination in this study was 21.17%. Mother's age educational status, and residence rural were significant predictors of age-appropriate vaccinations.

According to the findings from the current study age-appropriate vaccination coverage is very low (21.17 %) compared to the crude prevalence of child vaccination in Ethiopia. This finding is not supported by comparable literature where this figure is very low. The majority of the studies on this area are small samples and addressed children from limited study areas. Consequently, the age-appropriate vaccination coverage in this study is lower than the prevalence of study findings in El Salvador(26.7%) (29). Similarly, this finding is lesser than the timely vaccination of specific vaccine types in India where the coverage is 31%, and 34% for BCG and MCV (30). Generally, the very low finding of age-appropriate vaccination could be related to low economic status, low education, and other factors in Ethiopia (31-33).

In the present study, children of mothers aged 25-29, 35-39, and 40 and above were more likely to be vaccinated at an appropriate age compared to the children of mothers 15-19 years old during the data collection. This indicates that older mothers are at a better oddsof vaccinating their children in Ethiopia. This result is supported by the study conducted in China (34), and in Tanzania where younger mothers are at a risk of low age-appropriate vaccination (35). This could be because older mothers can have valuable experience in child caring they have likely raised children before, and they can have more exposure to health facilities and health information that can be delivered there (36, 37). On the other hand, mothers aged above 35 years

were less likely to vaccinate their children on time (38). Scientific research established that both extremities mother's age affects child caring (37).

Moreover, children of more educated mothers were more likely to be vaccinated at appropriate age. The children of mothers who have primary, secondary, and higher education had two times higher odds of being vaccinated compared to children from mothers who did not attend formal education. This study finding is supported by the study from China (34), Saudi Arabia (39), Tanzania (35), Cameron (40), and Ethiopia (41). This significance among educated mothers may be because of educated mothers being able to have awareness of the benefits of child vaccination. Also, educated mothers can be better at understanding the health care providers' counseling and messages on vaccination during awareness creation sessions at health facilities and at the community level many times the mothers get childcare information from health extension workers and women development armies in Ethiopia (42, 43). In support of this, scientific research has confirmed that children of educated mothers have a higher rate of complete immunization. This is because maternal education leads to better literacy skills and health-seeking behavior, which in turn improves immunization uptake for their children (44, 45).

Furthermore, children of mothers who live in rural areas were less likely vaccinated at the appropriate age compared to those who live in rural areas. This finding is supported by other studies in Nigeria(46) and Kenya(47). This could be due to various factors like limited access to health care services, inadequate infrastructure, and lower educational levels in rural settings. This implies that the government has to look into another strategic direction like community engagement and targeted intervention mechanisms to minimize rural-urban disparities.

Despite the current analysis being based on the most recent nationally representative survey data and usinga multilevel logistic regression to handle the variation within the level due to the

hierarchical nature of the EMDHS data, it is not free of limitation. One of the limitations of the study is the difficulty of showing temporal association because of the cross-sectional nature of the study. Another limitation, since the EMDHS survey is based on the participants' reports, there might be a recall bias.

Conclusion

From this study, we concluded that the prevalence of age-appropriate vaccination of children in Ethiopia is significantly below the desired level. Factors such as mother's age, education, and residence were significant predictors of age-appropriate vaccinations. Hence, we recommend that the stakeholders should give due attention to promote timely vaccination of children for their age.

Acknowledgment

The authors would like to express gratitude to www.DHSmeasure.orgfor granting access to the datasets.

References

1. Dang N, Liu T, Prow T. Nano-and microtechnology in skin delivery of vaccines. Micro and Nanotechnology in Vaccine Development: Elsevier; 2017. p. 327-41.
2. Gessner BD, Kaslow D, Louis J, Neuzil K, O'Brien KL, Picot V, et al. Estimating the full public health value of vaccination. Vaccine. 2017;35(46):6255-63.
3. Mast EE, Cochi SL, Kew OM, Cairns KL, Bloland PB, Martin R. Fifty Years of Global Immunization at CDC, 1966-2015. Public Health Reports. 2017;132(1):18-26.
4. Dheresa M, Dessie Y, Negash B, Balis B, Getachew T, Mamo Ayana G, et al. Child vaccination coverage, trends and predictors in Eastern Ethiopia: Implication for sustainable development goals. Journal of Multidisciplinary Healthcare. 2021:2657-67.
5. Organization WH. GIVS: global immunization vision and strategy: 2006-2015. World Health Organization, 2005.
6. Jain S, Basavaraj P, Singh S, Singla A, Kundu H, Singh K. Polio Eradication–Lessons from the Past and Future Perspective. Journal of clinical and diagnostic research: JCDR. 2014;8(7):ZC56.
7. _Ethiopia MoH. National Implementation Guideline for Expanded Program on Immunization. Available from http://repository.iifphc.org/bitstream/handle/123456789/1681/National-Implementation-Guidline-for-Expanded-Program-on-Immunization.pdf?sequence=1&isAllowed=y.%20June%202021.%208.Jimma%20MS,%20GebreEyesus%20FA,%20Chanie%20ES,%20Delelegn%20MWJPH,%20Medi. 2021.
8. Jimma MS, GebreEyesus FA, Chanie ES, Delelegn MW. Full vaccination coverage and associated factors among 12-to-23-Month Children at Assosa Town, Western Ethiopia, 2020. Pediatric Health, Medicine and Therapeutics. 2021:279-88.
9. Robinson CL. Advisory Committee on Immunization Practices recommended immunization schedules for persons aged 0 through 18 years—United States, 2016. American Journal of Transplantation. 2016;16(6):1928-9.
10. Doherty M, Buchy P, Standaert B, Giaquinto C, Prado-Cohrs D. Vaccine impact: Benefits for human health. Vaccine. 2016;34(52):6707-14.
11. Bloom DE, Canning D, Weston M. The value of vaccination. Fighting the diseases of poverty: Routledge; 2017. p. 214-38.
12. People H. Immunizations and infectious diseases. Washington, DC: US Department of Health and Humans Services. 2020.
13. Agenda WI. 2030: a global strategy to leave no one behind. World Health Organization. 2020;1.
14. UNICEF. The State of The World's Children 2023. For Every Child, Vaccination. UNICEF Innocenti—Global Office of Research and Foresight, Florence. 2023.
15. Gilbert Ulep V, Uy J. Too early, too late: Timeliness of child vaccination in the Philippines. PIDS Discussion Paper Series, 2019.
16. Mutua MK, Mohamed SF, Porth JM, Faye CM. Inequities in on-time childhood vaccination: evidence from Sub-Saharan Africa. American Journal of Preventive Medicine. 2021;60(1):S11-S23.
17. Dejene H, Girma D, Geleta LA, Legesse E. Vaccination timeliness and associated factors among children aged 12–23 months in Debre Libanos district of North Shewa Zone, Oromia Regional State, Ethiopia. Frontiers in Pediatrics. 2022;10:867846.
18. Marefiaw TA, Yenesew MA, Mihirete KM. Age-appropriate vaccination coverage and its associated factors for pentavalent 1-3 and measles vaccine doses, in northeast Ethiopia: A community-based cross-sectional study. PloS one. 2019;14(8):e0218470.
19. Woldu Anbesu E, Abubeker SA, Berhe BM. Age-appropriate vaccination practice and associated factors among mothers of children aged less than one year in the pastoral community of Afar region, Ethiopia. Human Vaccines & Immunotherapeutics. 2021;17(9):3178-85.

20. Biset G, Woday A, Mihret S, Tsihay M. Full immunization coverage and associated factors among children age 12-23 months in Ethiopia: systematic review and meta-analysis of observational studies. Human Vaccines & Immunotherapeutics. 2021;17(7):2326-35.
21. Abatemam H, Wordofa MA, Worku BT. Missed opportunity for routine vaccination and associated factors among children aged 0–23 months in public health facilities of Jimma Town. PLOS Global Public Health. 2023;3(7):e0001819.
22. Tilahun B, Mekonnen Z, Sharkey A, Shahabuddin A, Feletto M, Zelalem M, et al. What we know and don't know about the immunization program of Ethiopia: a scoping review of the literature. BMC Public Health. 2020;20(1):1-14.
23. Ethiopia MoHo. National Implementation Guideline for Expanded Program on Immunization. Avaialble at: http://repository.iifphc.org/bitstream/handle/123456789/1681/National-Implementation-Guidline-for-Expanded-Program-on-Immunization.pdf?sequence=1&isAllowed=y#:~:text=The%20Ethiopian%20National%20Expanded%20Program,mothers%20from%20vaccine%2Dpreventable%20diseases. June 2021
24. Ethiopia Moh. Health Sector Transformation Plan II 2020/21-2024/25. Avaialble on:https://faolex.fao.org/docs/pdf/eth208173.pdf. February 2021.
25. Indicators K. Mini demographic and health survey. EPHI and ICF. 2019.
26. Unicef. Children in Ethiopia.The situation of children in Ethiopia. Available from: https://www.unicef.org/ethiopia/children-ethiopia#:~:text=Ethiopia%20is%20home%20to%20about,growing%20economies%20in%20the%20world. 2018.
27. Institute EPH, ICF. Ethiopia mini demographic and health survey 2019: key indicators. Rockville, Maryland, USA. 2019.
28. Organization WH. Immunization, Vaccines and Biologicals. Avaialble from: https://www.who.int/teams/immunization-vaccines-and-biologicals/policies/who-recommendations-for-routine-immunization---summary-tables. Upadated March, 20223.
29. Suárez-Castaneda E, Pezzoli L, Elas M, Baltrons R, Crespin-Elías EO, Pleitez OAR, et al. Routine childhood vaccination programme coverage, El Salvador, 2011—In search of timeliness. 2014;32(4):437-44.
30. Shrivastwa N, Gillespie BW, Lepkowski JM, Boulton MLJTPIDJ. Vaccination timeliness in children under India's universal immunization program. 2016;35(9):955-60.
31. World Bank Group IEG. Ethiopia Country Program Evaluation (Approach Paper). 2024.
32. Ethiopian Public Health Institute and ICF. Mini demographic and health survey; Key indicators 2019.
33. Ali HA, Hartner A-M, Echeverria-Londono S, Roth J, Li X, Abbas K, et al. Vaccine equity in low and middle income countries: a systematic review and meta-analysis. 2022;21(1):1-30.
34. Hu Y, Chen Y, Guo J, Tang X, Shen LJHv, immunotherapeutics. Completeness and timeliness of vaccination and determinants for low and late uptake among young children in eastern China. 2014;10(5):1408-15.
35. Nadella P, Smith ER, Muhihi A, Noor RA, Masanja H, Fawzi WW, et al. Determinants of delayed or incomplete diphtheria-tetanus-pertussis vaccination in parallel urban and rural birth cohorts of 30,956 infants in Tanzania. 2019;19(1):1-11.
36. Camberis AL, McMahon CA, Gibson FL, Boivin JJI. Maternal age, psychological maturity, parenting cognitions, and mother–infant interaction. 2016;21(4):396-422.
37. Duncan GJ, Lee KT, Rosales-Rueda M, Kalil AJD. Maternal age and child development. 2018;55(6):2229-55.
38. Mekonnen ZA, Gelaye KA, Were MC, Tilahun BJBph. Timely completion of vaccination and its determinants among children in northwest, Ethiopia: a multilevel analysis. 2020;20:1-13.

39. Alrowaili GZ, Dar UF, Bandy AHJJof, medicine c. May we improve vaccine timeliness among children? A cross sectional survey in northern Saudi Arabia. 2019;26(2):113.
40. Chiabi A, Nguefack FD, Njapndounke F, Kobela M, Kenfack K, Nguefack S, et al. Vaccination of infants aged 0 to 11 months at the Yaounde Gynaeco-obstetric and pediatric hospital in Cameroon: how complete and how timely? 2017;17(1):1-7.
41. Woldu Anbesu E, Abubeker SA, Berhe BMJHV, Immunotherapeutics. Age-appropriate vaccination practice and associated factors among mothers of children aged less than one year in the pastoral community of Afar region, Ethiopia. 2021;17(9):3178-85.
42. Negussie A, Girma GJBHSR. Is the role of Health Extension Workers in the delivery of maternal and child health care services a significant attribute? The case of Dale district, southern Ethiopia. 2017;17:1-8.
43. Damtew ZA, Karim AM, Chekagn CT, Fesseha Zemichael N, Yihun B, Willey BA, et al. Correlates of the Women's Development Army strategy implementation strength with household reproductive, maternal, newborn and child healthcare practices: a cross-sectional study in four regions of Ethiopia. 2018;18(1):5-17.
44. Balogun SA, Yusuff HA, Yusuf KQ, Al-Shenqiti AM, Balogun MT, Tettey PJTPAMJ. Maternal education and child immunization: the mediating roles of maternal literacy and socioeconomic status. 2017;26.
45. Veneman AMJUc. Education is key to reducing child mortality. 2007;44(4):33-4.
46. Sibeudu FT, Uzochukwu BS, Onwujekwe OE. Rural–urban comparison of routine immunization utilization and its determinants in communities in Anambra states, Nigeria. SAGE open medicine. 2019;7:2050312118823893.
47. Allan S, Adetifa IM, Abbas K. Inequities in childhood immunisation coverage associated with socioeconomic, geographic, maternal, child, and place of birth characteristics in Kenya. BMC infectious diseases. 2021;21(1):553.
