# Supplementary material for: Age-appropriate vaccination and associated factors among children aged 12–35 months in Ethiopia: A multi-level analysis
Source: PLoS One. 2024 Oct 10;19(10):e0305801. doi: 10.1371/journal.pone.0305801 (PMC11466417; doi:10.1371/journal.pone.0305801)
Supplement: Attachment — Submitted filename: Response to Reviewers 1.docx [file pone.0305801.s003.docx]

**Response to Reviewers**

Paper ID: PONE-D-24-22259R1

Title: Age-appropriate Vaccination and Associated Factors among Children Aged 12- 35 Months in Ethiopia: A Multi-Level Analysis

Journal: PLOS ONE

Dear editor and reviewers,
Their insightful assessment of our work is really appreciated. Our work has improved as a result of these recommendations. After considering the advice, we have revised our work. The problems are addressed point by point in the following manner, with the answers indicated in blue.

Please include the following items when submitting your revised manuscript:

- A rebuttal letter that responds to each point raised by the academic editor and reviewer(s). You should upload this letter as a separate file labeled 'Response to Reviewers'
  - Included
- A marked-up copy of your manuscript that highlights changes made to the original version. You should upload this as a separate file labeled 'Revised Manuscript with Track Changes'.
  - Included
- An unmarked version of your revised paper without tracked changes. You should upload this as a separate file labeled 'Manuscript'.
  - Included

Journal Requirements:

Please review your reference list to ensure that it is complete and correct. If you have cited papers that have been retracted, please include the rationale for doing so in the manuscript text, or remove these references and replace them with relevant current references. Any changes to the reference list should be mentioned in the rebuttal letter that accompanies your revised manuscript. If you need to cite a retracted article, indicate the article’s retracted status in the References list and also include a citation and full reference for the retraction notice.

Dear editor, thank you for your reflections.

We removed one reference and replaced with other. The reference was removed because it was achieved

- **Removed reference:**

People H. Immunizations and infectious diseases. Washington, DC: US Department of Health and Humans Services. 2020.

- **Added reference:**

Talbird SE, Carrico J, La EM, Carias C, Marshall GS, Roberts CS, et al. Impact of routine childhood immunization in reducing vaccine-preventable diseases in the United States. Pediatrics. 2022;150(3):e2021056013. <https://doi.org/10.1542/peds.2021-056013>

- In addition, for each reference we included
- DOI if the article as DOI number and
- URL if it has no DOI

Then we highlighted with yellow color on URL and DOI to indicate change made

- Moreover, duplicated references which were cited with different numbers have been corrected by removing the duplicates. Here are the details:

1. **T****he following two references were the same same, and one has been remved**

- 7.	Ethiopia MoH. National Implementation Guideline for Expanded Program on Immunization. Available from <http://repository.iifphc.org/bitstream/handle/123456789/1681/National-Implementation-Guidline-for-Expanded-Program-on-Immunization.pdf?sequence=1&isAllowed=y.%20June%202021.%208.Jimma%20MS,%20GebreEyesus%20FA,%20Chanie%20ES,%20Delelegn%20MWJPH,%20Medi>. 2021.
- 23.	Ethiopia MoHo. National Implementation Guideline for Expanded Program on Immunization. Avaialble at: <http://repository.iifphc.org/bitstream/handle/123456789/1681/National-Implementation-Guidline-for-Expanded-Program-on-Immunization.pdf?sequence=1&isAllowed=y#:~:text=The%20Ethiopian%20National%20Expanded%20Program,mothers%20from%20vaccine%2Dpreventable%20diseases>. June 2021

**Corrected as:**

7.	Minister Of Health-Ethiopia. National Implementation Guideline for Expanded Program on Immunization. June, 2021. Available from: http://196.189.110.22/bitstream/handle/123456789/1681/National-Implementation-Guidline-for-Expanded-Program-on-Immunization.pdf?sequence=1&isAllowed=y

2. **The following three references were the same, and we removed two and corrected the remaining one**

- 25.	Indicators K. Mini demographic and health survey. EPHI and ICF. 2019.
- 27.	Institute EPH, ICF. Ethiopia mini demographic and health survey 2019: key indicators. Rockville, Maryland, USA. 2019.
- 32.	Ethiopian Public Health Institute and ICF. Mini demographic and health survey; Key indicators 2019.

**Corrected as:**

24.	Ethiopian Public Health Institute (EPHI) [Ethiopia] and ICF. 2021. Ethiopia Mini Demographic and Health Survey 2019: Final Report. Rockville, Maryland, USA: EPHI and ICF. Available from: https://dhsprogram.com/pubs/pdf/FR363/FR363.pdf

3. **The following two references were the same, and one has been removed**

- **Correct one:** 19.	Woldu Anbesu E, Abubeker SA, Berhe BM. Age-appropriate vaccination practice and associated factors among mothers of children aged less than one year in the pastoral community of Afar region, Ethiopia. Human Vaccines & Immunotherapeutics. 2021;17(9):3178-85.
- 41.	Woldu Anbesu E, Abubeker SA, Berhe BMJHV, Immunotherapeutics. Age-appropriate vaccination practice and associated factors among mothers of children aged less than one year in the pastoral community of Afar region, Ethiopia. 2021;17(9):3178-85.

**Reviewer 1**

Line/s:
13: mothers aged (remove d)

Thank you reviewer for your concern, we removed letter d
28: rotavirus and rotavirus (duplication)

Thank you reviewer for your feedback, we removed the duplication

38/42: vaccine-prevalent (preventable)

Thank you, reviewer, for your concern. We repacked prevalent to preventable
43: Nowadays (use different phrase - currently, at present, at time of writing)

Thank you for your suggestions, we replaced with currently
85: Children were excluded if the dates of vaccination were inconsistent with their birth dates. (Please reword to add clarity)

Thank you for your concern, we rephrased as children were excluded if their vaccination dates didn’t align with their birth dates.
33-34 &105-106 & 112, 113 - specify content of pentavalent vaccine (consistency)

Thank you for your suggestions. We tried to make consistent especially on line 105-106 & 112, 113 but regarding the concepts on 33-34, the concept of Penta was not initiated at that moment.
148: The mean age of mothers was a 28.58 ±6.27 year (remove 'a' and add years)

Thank you reviewer for your concern, we removed letter a and added years
155: Table 1 - abbreviation not explained - "Sex of HH++ head" (add abbreviation to bottom of table)

Thank you for your suggestions. Now its full name is included under the table: HH = Head of Household
229: specify abbreviation earlier (e.g. line 38) (VPD - introduce abbreviation in text earlier)

Thank you for your suggestions. VPD –now it is included in text

**Reviewer #2:** The changes made have enhanced the quality of the manuscript. Just two very minor editorial errors can still be corrected;
Dear reviewer thank you for your inspiration.
Line 3 in the abstract: public health measure should be singular, not plural
Thank you for your concern. Now it is corrected to public health measure
Line 35: should be the target not a target.

Thank you for the suggestion. We removed a and replaced by the
